# Design of Small Non-Peptidic Ligands That Alter Heteromerization between Cannabinoid CB_1_ and Serotonin 5HT_2A_ Receptors

**DOI:** 10.1021/acs.jmedchem.4c01796

**Published:** 2024-12-27

**Authors:** Minos-Timotheos Matsoukas, Marc Ciruela-Jardí, Maria Gallo, Sergi Ferre, David Andreu, Vicent Casadó, Leonardo Pardo, Estefanía Moreno

**Affiliations:** † Department of Biomedical Engineering, University of West Attica, Ag. Spyridonos, Egaleo 12243, Greece; ‡ Laboratori de Medicina Computacional, Unitat de Bioestadística, Facultat de Medicina, Universitat Autónoma de Barcelona, Bellaterra 08193, Spain; § Department of Biochemistry and Molecular Biomedicine, Faculty of Biology, Institute of Biomedicine of the University of Barcelona (IBUB),University of Barcelona, Barcelona 08028, Spain; ∥ Department of Medicine and Life Sciences (MELIS-UPF), Universitat Pompeu Fabra, Barcelona 08003, Spain; ⊥ Integrative Neurobiology Section, National Institute on Drug Abuse, Intramural Research Program, National Institutes of Health, Baltimore, Maryland 21224, United States

## Abstract

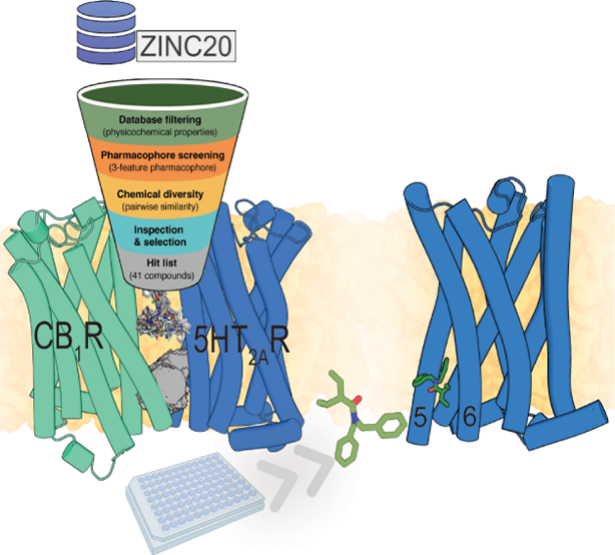

Activation of cannabinoid CB_1_ receptors (CB_1_R) by agonists induces analgesia but also induces cognitive impairment through the heteromer formed between CB_1_R and the serotonin 5HT_2A_ receptor (5HT_2A_R). This side effect poses a serious drawback in the therapeutic use of cannabis for pain alleviation. Peptides designed from the transmembrane helices of CB_1_R, which are predicted to bind 5HT_2A_R and alter the stability of the CB_1_R-5HT_2A_R heteromer, have been shown to avert CB_1_R agonist-induced cognitive impairment while preserving analgesia. Using these peptides as templates, we have now designed nonpeptidic small molecules that prevent CB_1_R-5HT_2A_R heteromerization in bimolecular fluorescence complementation assays and the heteromerization-dependent allosteric modulations in cell signaling experiments. These results provide proof-of-principle for the design of optimized ligand-based disruptors of the CB_1_R-5HT_2A_R heteromer, opening new perspectives for *in vivo* studies.

## Introduction

Cannabinoid agonists have proven their analgesic potential in clinical studies of cancer pain,^1,2^ neuropathic pain,^3^ and other types of pain. The effects of cannabinoids are primarily mediated through the cannabinoid CB_1_ receptor (CB_1_R) and CB_2_ receptor (CB_2_R). CB_1_R is abundant in the central nervous system, whereas CB_2_R is mainly expressed in the immune system.^4^ However, the therapeutic response elicited by cannabinoid agonists such as delta-9-tetrahydrocannabinol (THC), the principal psychoactive constituent of the *Cannabis sativa* plant (more commonly known as marijuana), is hampered by side effects, namely memory impairment.^5^ Recent findings linked the cognitive side effects of THC to complexes of CB_1_R and serotonin 5-HT_2A_ receptor (5HT_2A_R).^6^ CB_1_R and 5HT_2A_R belong to the G protein-coupled receptor (GPCR) family, which frequently forms oligomers in cells.^7^ Importantly, the formation of the CB_1_R-5HT_2A_R heteromer has been characterized by several techniques, including *in situ* proximity ligation assays and mice expressing heteromerization-deficient receptors,^6^ which strongly support their existence in native tissues.^8^ Moreover, it was also shown that the expression of the CB_1_R-5HT_2A_R heteromer was enhanced in olfactory neuroepithelium cells of cannabis users^9^ and schizophrenia patients.^10^

The use of synthetic peptides with the sequence of the transmembrane (TM) domains of the receptor fused to the cell-penetrating HIV transactivator of transcription (Tat) peptide (GRKKRRQRRR)^11^ has been very valuable in predicting contacts among TMs of interacting GPCRs.^12−15^ Using this approach, we have previously identified that synthetic peptides TM5-Tat and Tat-TM6 (but not TM7-Tat), derived from CB_1_R, were able to decrease bimolecular fluorescence complementation (BiFC) signals elicited by receptors fused to two complementary halves of YFP (5HT_2A_R-cYFP and CB_1_R-nYFP), which suggests that the arrangement of CB_1_R and 5HT_2A_R protomers in the heteromer is via TMs 5 and 6 (TM5/6 interface).^6^ More importantly, interfering TM5-Tat and Tat-TM6, but not TM7-Tat, peptides led to a selective abrogation of memory impairments caused by exposure to THC *in vivo*.^6^

It was thus reasonable to assume that altering the formation of the CB_1_R-5HT_2A_R heteromer might be an effective strategy for harnessing the therapeutic potential of THC while avoiding its side effects. Accordingly, we recently developed a drug-like 16-residue peptide, displaying 9 key amino acids from the 37-residue TM5-Tat peptide, which could penetrate the blood–brain barrier via a cell-penetrating sequence of 7 amino acids.^16^ Following up on these results, we now report the design of small nonpeptidic molecules capable of mimicking the interaction of the peptides with 5HT_2A_R and, thus, capable of disrupting the CB_1_R-5HT_2A_R heteromer. As previously proposed, a heteromer-disrupting ligand would allow the use of cannabis to fight pain while avoiding cognitive side effects. This is relevant because it has been suggested that cannabinoid ligands could fill the therapeutic gap between opioids and nonsteroidal anti-inflammatories in multiple moderate pain conditions.^17^

## Results

### VYAYMYILW^5.63^-Tat is the Shortest Peptide That Alters CB_1_R-5HT_2A_R Heteromerization as Efficiently as TM5-Tat

**Figure 1:**
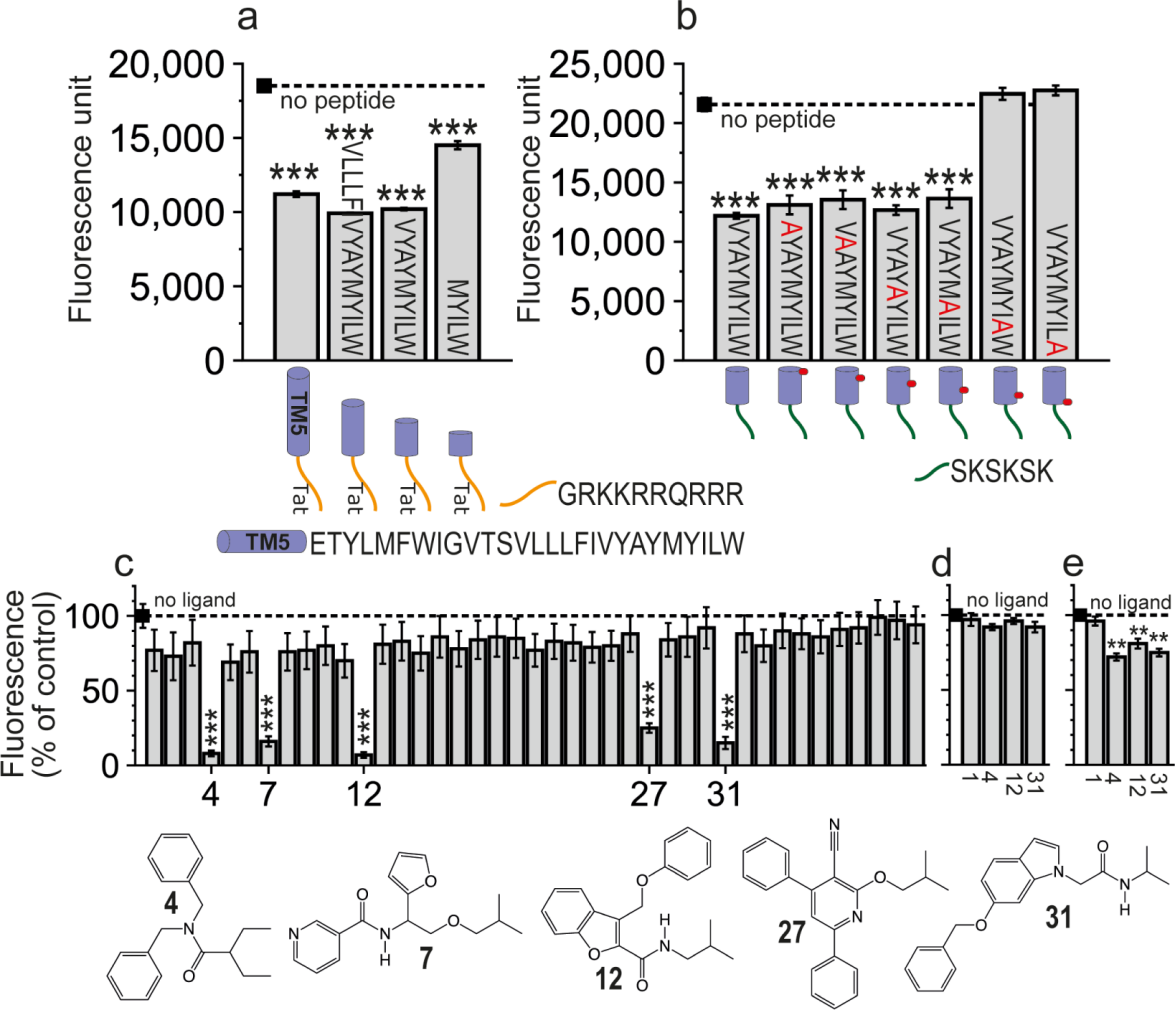
BiFC analysis of the effect of (a and b) designed peptides or (c) 41 nonpeptidic ligands on (a–c) CB_1_R-5HT_2A_R heteromerization and (d) CB_1_R-CB_1_R or (e) 5HT_2A_R-5HT_2A_R homomerization. Fluorescence (530 nm) of HEK-293T cells transfected with (a–c) 5HT_2A_R-cYFP and CB_1_R-nYFP, (d) CB_1_R-cYFP and CB_1_R-nYFP, and (e) 5HT_2A_R-cYFP and 5HT_2A_R-nYFP, treated with vehicle (black square, dashed line) or 4 μM peptide for 4 h (a and b) or 4 μM ligand for 4 h (c–e). Values in panels c–e represent fluorescence relative to control (no ligand, 100%). Values are mean ± SEM of *n* = 6–10. One-way ANOVA followed by Dunnett’s multiple comparison post hoc test was used for statistical analysis versus the control condition (black squares, no peptide, or no ligand) (**p < 0.001; ***p < 0.0001). Chemical structures of compounds **4**, **7**, **12**, **27**, and **31**, which significantly decreased fluorescence, are shown.

We had previously identified the TM5-Tat peptide, derived from TM 5 of CB_1_R, that interacts with TMs 5 and 6 of 5HT_2A_R and alters CB_1_R-5HT_2A_R heteromerization.^6^ Here, we wanted to delineate the minimal region of the 37-residue TM5-Tat peptide (27 amino acids from CB_1_R plus 10 from Tat) that is capable of interacting with 5HT_2A_R. Therefore, we tested a variety of shorter peptides, spanning the TM5-Tat peptide, to alter BiFC signals between 5HT_2A_R-cYFP and CB_1_R-nYFP in HEK-293T cells (Figure 1a). We have designed the ^5.49^VLLLFIVYAYMYILW^5.63^-Tat, ^5.55^VYAYMYILW^5.63^-Tat, and ^5.59^MYILW^5.63^-Tat peptides using our previous MD simulations of TM5-Tat in complex with 5HT_2A_R.^16^ The residues that form the peptides will be named using the one-letter code of the amino acids and the Ballesteros–Weinstein nomenclature^18^ of the CB_1_R sequence as a superscript. The peptides are three, five, and six helical turns shorter, respectively, than the reference TM5-Tat peptide. All caused a clear decrease in BiFC signals, though the shortest one, ^5.59^MYILW^5.63^-Tat, being less effective than TM5-Tat or the other two peptides (Figure 1a).

### LW^5.63^ Are the Residues Contributing More Significantly to 5HT_2A_R Binding

In protein–protein interactions, “hot spots” are amino acids that contribute more significantly to the binding affinity. To experimentally probe the key molecular interactions of the minimal VYAYMYILW^5.63^-Tat peptide with 5HT_2A_R, we mutated the amino acids predicted to interact with 5HT_2A_R to Ala (Figure 1b). Since the 6-residue SKSKSK^19^ cell-penetrating sequence can replace Tat with similar effects,^16^ the experiments were performed with SKSKSK-extended peptides (Figure 1b). Both the L^5.62^ (VYAYMYIAW^5.63^-SKSKSK) and W^5.63^ (VYAYMYILA^5.63^-SKSKSK) mutants were unable to decrease fluorescence; hence, residues LW^5.63^ were deemed key for the interaction with 5HT_2A_R.

### A 3-Point Pharmacophore Model for the Interaction with 5HT_2A_R

**Figure 2:**
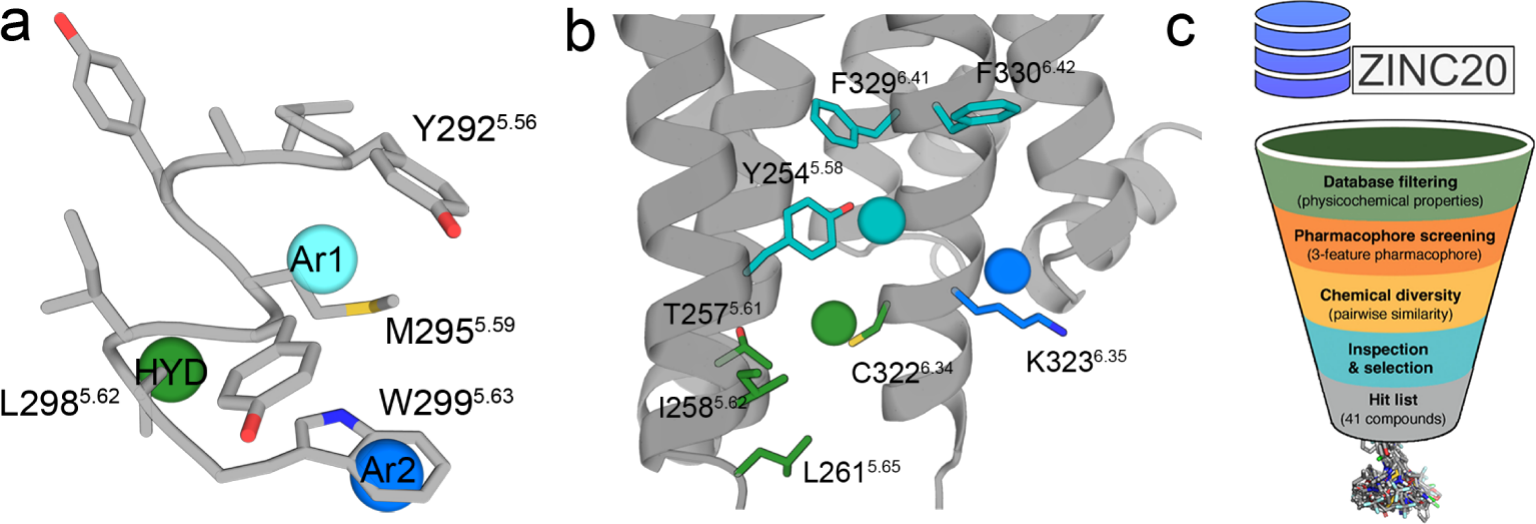
A pharmacophore model for the interaction with 5HT_2A_R. (a) Molecular model of the ^5.55^VYAYMYILW^5.63^ peptide (gray sticks), derived from TM 5 of CB_1_R, and the 3-point pharmacophore containing an aromatic feature (Ar1, blue circle) positioned over W^5.63^ of the peptide, a hydrophobic feature (HYD, green circle) positioned over L^5.62^ of the peptide, and an additional aromatic feature (Ar2, cyan circle) positioned approximately between Y^5.56^ and M^5.59^ of the peptide. (b) Position of this 3-point pharmacophore relative to our previously reported 5HT_2B_R-based homology model of 5HT_2A_R.^6,16^ In this homology model, Ar1 is predicted to interact with Lys323^6.35^ and Phe330^6.42^ side chains (blue/cyan sticks), HYD with Thr257^5.61^, Ile258^5.62^, Leu261^5.65^, and Cys321^6.34^ side chains (green sticks), and Ar2 (cyan circle) with Tyr254^5.58^, Phe329^6.41^, and Phe330^6.42^ side chains (cyan sticks). (c) The virtual screening process from the ZINC database^20^ involved the steps of physicochemical properties calculation and filtering, conformer generation and pharmacophore screening, chemical diversity calculation of the top-ranked compounds, and inspection and final selection of the molecules to be purchased and tested.

We aimed to develop a model of a pharmacophore that embodies the structural features necessary for 5HT_2A_R recognition. We inferred 3-point pharmacophoric features (Figure 2a) based on the above experimental results with different peptides (Figure 1a,b) and the previously reported computational model between TM5-Tat and 5HT_2A_R (Figure 2b).^16^ The model includes first an aromatic feature (Ar1) that mimics the key W^5.63^ side chain identified in the Ala scan (Figures 1b, 2a) predicted to interact with Lys323^6.35^ and Phe330^6.42^ of 5HT_2A_R (Figure 2b) (the 5HT_2A_R residues will be named using the three-letter amino acid code to distinguish them from the peptide residues). Second, a hydrophobic feature (HYD) mimicking the L^5.62^ side chain of the peptide (Figure 2a) that would be in a small membrane-facing site between TMs 5 and 6 of 5HT_2A_R, flanked by Thr257^5.61^, Ile258^5.62^, Leu261^5.65^, and Cys321^6.34^ of 5HT_2A_R (Figure 2b). Third, an additional aromatic feature (Ar2) would interact with the Tyr254^5.58^, Phe329^6.41^, and Phe330^6.42^ cluster of aromatic rings of 5HT_2A_R (Figure 2b). The rationale for adding this aromatic feature, placed approximately between Y^5.56^ and M^5.59^ of the peptide (Figure 2a), is that the removal of a helical turn (VYAY) in the ^5.55^VYAYMYILW^5.63^-Tat peptide makes the resulting ^5.59^MYILW^5.63^-Tat peptide clearly less efficient in decreasing BiFC signals than TM5-Tat (Figure 1a).

### A Pharmacophore-Based Virtual Screening Identified 41 Small Nonpeptidic Ligands That Potentially Bind 5HT_2A_R and Alter CB_1_R-5HT_2A_R Heteromerization

Approximately 12 M compounds of the Clean Drug-like subset of the ZINC database^20^ were filtered to obtain the screening subset. Since the binding site is in the hydrophobic membrane environment, the criteria for filtering the library were Lipinski’s rules (maximum 5 hydrogen bond donors, 10 hydrogen bond acceptors, molecular weight of 500 Da, and log *P* of 5)^21^ slightly modified for this specific task. Explicitly, the molecular weight limits were set at 250–500 Da, the maximum hydrogen bond donors (<4) and acceptors (<8) were decreased to reduce polarity, log *P* was increased to the 2–7 range to favor hydrophobicity, and the number of aromatic groups in the ligand was set to ≥2 to meet the pharmacophore requirements. The initial database was filtered using Openbabel,^22^ leading to a subset of ∼4 M compounds (see Methods). Diverse conformations (250 per compound) of these ∼4 M compounds were rigid body-fitted (no additional conformational changes in the ligand were allowed) to the 3-point pharmacophore model. These were ranked from best to worst fitting (see Methods), and ∼300 k putative disruptors were mapped again onto the pharmacophore model using a flexible fit (additional conformational changes in the ligand are allowed). Finally, top scoring ∼4 k compounds (see Methods) were visually inspected for chemical complementation between the ligand and the predicted 5HT_2A_R cavity. These procedures (Figure 2c) finally yielded 41 diverse compounds (Table S1) that were selected for purchasing and experimental validation.

### Five Compounds Alter CB_1_R-5HT_2A_R Heteromerization in BiFC Assays

The 41 small nonpeptidic compounds were tested in BiFC assays (Figure 1c) for their ability to alter CB_1_R-5HT_2A_R heteromerization (see Methods). Five of the 41 compounds (12% hit rate) caused a significant decrease of BiFC signals, like the reported peptides. All five structures (Figure 1c) contain at least two aromatic rings, phenyl and/or aromatic heterocycles, and a branched hydrophobic group. Among these, compounds **4**, **12**, and **31**, causing the largest decrease in fluorescence, were selected for further analysis. Inactive compound **1** (negative control) and active compounds **4**, **12**, and **31** were also tested to alter BiFC signals between CB_1_R-cYFP and CB_1_R-nYFP (Figure 1d) and between 5HT_2A_R-cYFP and 5HT_2A_R-nYFP (Figure 1e). These experiments show that compounds **4**, **12**, and **31**, but not compound **1**, bind to 5HT_2A_R, but not to CB_1_R, and influence the homodimerization of 5HT_2A_R.

### Compounds 4, 12, and 31 Block Cross-Antagonism and Negative Crosstalk Biochemical Signatures of the CB_1_R-5HT_2A_R Heteromer

**Figure 3:**
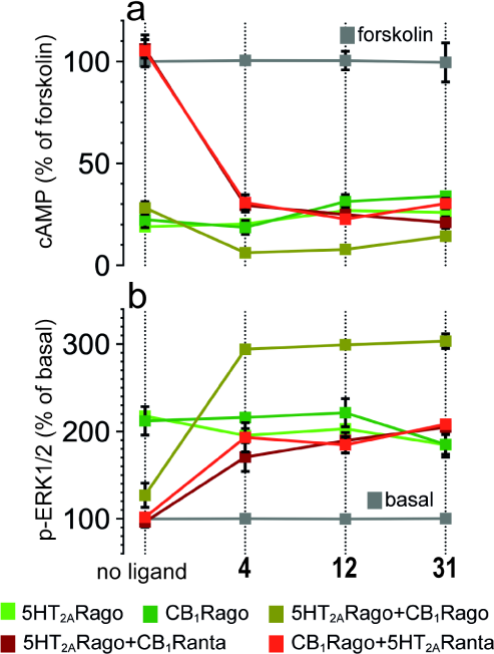
Effect of selected compounds on the decrease in FK (0.5 μM)-induced cAMP (a) or increase in ERK1/2 phosphorylation (b). Cells were preincubated with a vehicle (no ligand) or with the 5HT_2A_R agonist DOI (5HT_2A_Rago, 100 nM) or the CB_1_R agonist WIN (CB_1_Rago, 100 nM) or in combinations with the 5HT_2A_R antagonist MDL (5HT_2A_Ranta, 300 nM) (CB_1_Rago + 5HT_2A_Ranta) or the CB_1_R antagonist RIM (CB_1_Ranta, 1 μM) (5HT_2A_Rago + CB_1_Ranta), or both agonists (5HT_2A_Rago + CB_1_Rago). Quantification of phosphorylated ERK-1/2 was determined by the α-screen bead-based technology. Values in panel a are mean ± SEM of *n* = 6 of FK-treated cells, and values in panel b, expressed as a percentage of basal (nontreated cells), are mean ± SEM of *n* = 6. Two-way ANOVA followed by Tukey’s multiple comparison tests was used to analyze the data (Table S2).

We have shown that the formation of the CB_1_R-5HT_2A_R heteromer alters the signaling response compared to receptors expressed individually on the cell surface.^6,9,10,16,23^ Heteromer formation triggers a switch in the G-protein coupling for 5HT_2A_R from Gq to Gi proteins. Antagonist binding to one of the receptors blocks signaling of the interacting receptor (cross-antagonism), and costimulation with both agonists does not produce an additive effect (negative crosstalk) (see (24) for definition of these effects). Aiming to ascertain the biochemical signatures of the CB_1_R-5HT_2A_R heteromer in the presence of **4**, **12**, and **31**, we monitored receptor activation by measuring cAMP levels [decrease in forskolin (FK)-induced cAMP] or the increase in pERK in the ERK1/2 phosphorylation pathway. Cells stimulated with FK and treated with the CB_1_R agonist WIN 55,212-2 (WIN) or the 5HT_2A_R agonist 2,5-dimethoxy-4-iodoamphetamine (DOI) showed reduced levels of cAMP production in the absence and presence of compounds **4**, **12**, and **31** (Figure 3a). Hence, none of these compounds, neither the full-length TM5-Tat or Tat-TM6 peptides^6^ nor their shortened versions,^16^ influenced G protein coupling preferences (5HT_2A_R remained Gi-coupled). In the CB_1_R-5HT_2A_R heteromer, decreased cAMP or increased pERK caused by 5HT_2A_R agonist DOI is similar (no statistical differences) in the absence and presence of compounds **4**, **12**, and **31** (Figure 3). Thus, these compounds do not allosterically influence the activity of the orthosteric agonist DOI in 5HT_2A_R activation. Moreover, coadministration of WIN and DOI agonists does not lead to a further statistically significant decrease in cAMP or an increase in pERK (negative crosstalk) in the absence of the ligands. In contrast, in the presence of **4**, **12**, and **31**, simultaneous addition of both agonists causes a statistically significant cAMP decrease or pERK increase (absence of negative crosstalk), relative to single agonist administration (Figure 3).

The CB_1_R antagonist rimonabant (RIM) blocked the decrease in FK-induced cAMP or the increase in pERK triggered by the 5HT_2A_R agonist DOI, and the 5HT_2A_R antagonist MDL 100,907 (MDL) also blocked the decrease in cAMP and increase in pERK induced by the CB_1_R agonist WIN (bidirectional cross-antagonism). Cross-antagonism is due to the formation of a high surface complementarity between TMs 5 and 6 of the two protomers, via a four-helix bundle,^25^ which blocks the opening of the intracellular cavity for G protein binding at the other protomer.^7^ Notably, compounds **4**, **12**, and **31** eliminated this bidirectional cross-antagonism (Figure 3). Thus, in the presence of **4**, **12**, and **31**, the CB_1_R agonist WIN plus the 5HT_2A_R antagonist MDL and the 5HT_2A_R agonist DOI plus the CB_1_R antagonist (RIM) can decrease cAMP or increase pERK, as efficiently as the CB_1_R agonist WIN and the 5HT_2A_R agonist DOI alone, respectively. Because orthosteric antagonists do not signal on their own, cross-antagonism requires direct protein–protein interaction at the TM level. The fact that these compounds cancel the bidirectional cross-antagonism suggests that they change these pharmacological properties of the CB_1_R-5HT_2A_R heteromer by altering the heteromeric interface.

In summary, alteration of the heteromeric interface of the CB_1_R-5HT_2A_R heteromer with ligands did not block the coupling of 5HT_2A_R to Gi but abolished the reduced signaling upon simultaneous activation of both receptors (negative crosstalk) and the bidirectional cross-antagonism. This suggests that ligands that interfere with the heteromeric interface can remove the heteromer-dependent structural restrictions for receptor activation without disrupting heteromerization and its G protein-coupling preferences.

### Compounds 4, 12, and 31 Bind in a Membrane-Facing Site between TMs 5 and 6 of 5HT_2A_R

**Figure 4:**
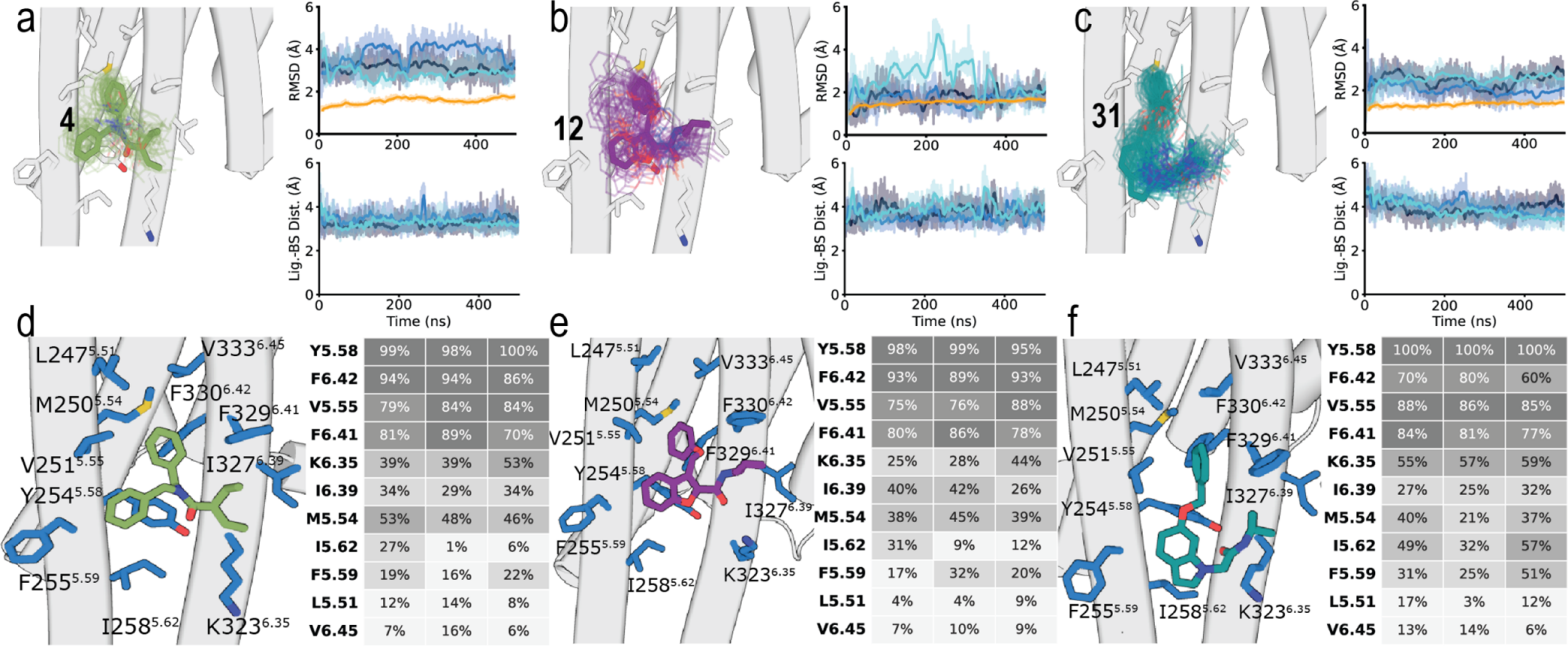
Computational model between compounds **4**, **12**, and **31** and 5HT_2A_R (PDB id 7WC4). (a–c) Representative structures (solid sticks) and evolution (lines) of compound **4** (a, in green), **12** (b, in purple), and **31** (c, in blue) in complex with 5HT_2A_R (gray cylinders, only the initial structure is shown) during MD simulations (one replica is displayed for better visualization although additional replicas showed consistent behavior). The stability of the ligand–receptor complexes (top panels) was analyzed via root mean-square deviations (RMSD) of the ligand heavy atoms as devised from three replicas of unbiased 500 ns MD simulations (blue colors), and the stability of 5HT_2A_R was analyzed via RMSD of the receptor C atoms (orange). Evolution of the distances between the center of mass (COM) of the ligand and the residues in the binding site (bottom panels), along the trajectories (blue colors). (d–f) Detailed views and heatmaps (calculated with GetContacts, https://getcontacts.github.io/interactions.html) depicting the predicted interactions between compounds **4** (d), **12** (e), and **31** (f) and 5HT_2A_R during three replicas of unbiased 500 ns MD simulations.

Our previously reported 5HT_2B_R-based homology model of 5HT_2A_R bound to the TM5-Tat peptide^16^ predicted that short peptides would bind in a small membrane-facing site between TMs 5 and 6 of 5HT_2A_R. Here, we have used the recently released structures of 5HT_2A_R^26,27^ to identify the binding mode of compounds **4**, **12**, and **31**. Among the multiple docking models (see Figure S1 and Methods), we have first selected poses in which the terminal phenyl ring of the ligands is placed between the group of aromatic rings of 5HT_2A_R formed by Tyr254^5.58^, Phe329^6.41^, and Phe330^6.42^. Second, we evaluated the stability of the binding mode using unbiased MD simulations (see Methods). Figure 4a-c shows root-mean-square deviations (RMSDs) of the heavy atoms of the ligands and the C atoms of 5HT_2A_R for simulations of docking poses that are stable and reproduced repeatedly. As expected, the RMSD values of the ligands (<4 Å) are higher than those of the protein (<2 Å) because, unlike orthosteric binding sites, the cavities of membrane-facing sites are formed in part by the surrounding, flexible lipids. Hence, compounds **4**, **12**, and **31** have 33%, 38%, and 32%, respectively, of the total solvent-accessible area (SASA of 511 Å^2^, 522 Å^2^, and 523 Å^2^, respectively) exposed to the membrane (MESA of 171 Å^2^, 197 Å^2^, and 167 Å^2^, respectively), calculated from three replicates of 500 ns of unbiased MD simulations. Moreover, the distance between the center of mass (COM) of compounds **4**, **12**, and **31** and the residues in the binding site, along the trajectories (Figure 4a-c), shows that all three ligands remained stable at the predicted binding site. Inactive compound **40**, used as a negative control, is less stable in the binding site than these active compounds **4**, **12**, and **31** in analogous MD simulations (Figure S2).

Figure 4d–f shows heatmaps of the predicted interactions of compounds **4**, **12**, and **31** with amino acids of the membrane-facing pocket between TMs 5 and 6 of 5HT_2A_R. The three ligands have very similar binding modes, interacting with the same side chains of 5HT_2A_R. First, the common terminal phenyl ring of the ligands maintains the initially modeled aromatic interactions with the group of aromatic rings of 5HT_2A_R formed by Tyr254^5.58^, Phe329^6.41^, and Phe330^6.42^. Additional interactions between this aromatic ring of the ligand and Met250^5.54^ and Val251^5.55^ are observed. Notably, the highly polarizable sulfur atom of Met can form stronger interactions, than aromatic or hydrophobic side chains, with aromatic groups.^28^ On the other side of the molecule, the branched hydrophobic group of the ligands interacts with Lys323^6.35^, Ile327^6.39^, and Phe330^6.42^. The central aromatic heterocycle of **12** and **31**, or the phenyl ring of **4**, interacts with Tyr254^5.58^, Phe255^5.59^, and Ile258^5.62^. Finally, the carbonyl groups of all three ligands form a hydrogen bond interaction with Tyr254^5.58^. This lipid-facing site between TMs 5 and 6 was labeled Orphan Site 5 (OS5) in the recent analysis of the pocketome of GPCRs.^29^

## Discussion and Conclusions

The analysis of the pocketome of GPCRs has shown multiple nonconserved pockets in addition to the orthosteric pocket, where endogenous ligands bind.^29^ Many of these sites had already been identified in structures of allosteric ligands with GPCRs. In contrast, other sites remained untargeted (orphan) sites. One of these orphan sites (OS5) is located at the membrane-facing part of 5HT_2A_R, between TMs 5 and 6, where compounds **4**, **12**, and **31** are predicted to bind. However, these compounds do not allosterically influence the activity of the orthosteric agonist DOI in 5HT_2A_R activation, so we cannot define this site as allosteric or these compounds as allosteric modulators. An alternative role of these membrane-facing cavities is to modulate the stability of the GPCR oligomers. The TM5/6 interface is particularly relevant because it is frequently predicted in GPCR heteromers^6,15,30^ and directly participates in the allosteric modulation of the partner receptor.^7^

The design of small molecules targeting protein–protein interfaces, and GPCR interfaces in particular, is specially challenging as they have large and flat contact areas without well-defined pockets, which would be “undruggable” according to the classical definition of druggable sites.^31^ Therefore, most ligands binding these pockets have a complex structure and are very lipophilic, as part of their binding cavity is promoted by the lipidic membrane.^32^ This makes their optimization to drug candidates more challenging.^33^ However, the field of designing molecular inhibitors^34^ or molecular glues^35^ to modulate protein–protein interactions with small molecules is rapidly growing.

We developed an approach to design molecular inhibitors of GPCR interactions. First, after characterizing a GPCR heteromer of interest,^6^ we designed synthetic peptides with the sequence of TM domains of one of the receptors fused to cell-penetrating peptides such as the Tat^11^ or SKSKSK^19^ sequence. The ability of these peptides to disrupt BiFC signals of the two receptors separately fused to the complementary cYFP and nYFP predicts contacts among TM domains of interacting GPCRs.^36^ The 37-residue-long TM5-Tat and 36-residue-long Tat-TM6 peptides, derived from the CB_1_R sequence, were able to disrupt BiFC signals between 5HT_2A_R-cYFP and CB_1_R-nYFP, suggesting a TM5/6 heteromeric interface.^6^ Second, by downsizing the length of the 37-residue TM5-Tat interfering peptide to only include amino acids essential for receptor interaction, we designed a fully druggable 16-mer that alters CB_1_R-5HT_2A_R heteromerization as efficiently.^16^ Third, Ala scanning mutagenesis identified “hot spots”, i.e., side chains that are more relevant than others for peptide-receptor interaction. And fourth, we designed small nonpeptidic molecules capable of mimicking the “hot spot” recognition elements with 5HT_2A_R. We accomplished this by performing pharmacophore-guided virtual screening, a well-established successful computational tool for rational drug design.^37^

The candidate compounds, selected *in silico* from chemical databases, required experimental validation. Typically, for orthosteric ligands, pharmacological assays are performed to measure affinity or/and efficacy. However, when targeting orphan sites, with no known ligands binding to this site and without allosteric effects on orthosteric agonists, alternative assays must be used. We tested the ability of these compounds to alter CB_1_R-5HT_2A_R heteromerization in BiFC assays, in which five of the 41 compounds (12% hit rate) caused a significant disruptive effect. More importantly, we also tested whether these compounds counteract the heteromerization-dependent allosteric effects that CB_1_R ligands exert on 5HT_2A_R ligands and vice versa.^7,24^ In agreement, compounds **4**, **12**, and **31** blocked part of the allosteric effects within the CB_1_R-5HT_2A_R heteromer. In fact, the presence of these compounds counteracted the previously described CB_1_R-5HT_2A_R heteromer-dependent negative crosstalk and cross-antagonism.^6^ However, these ligands did not influence G protein coupling preferences, as 5HT_2A_R remained Gi-coupled. This suggests that other domains of CB_1_R (intracellular loops or/and C-terminal) might contribute to the Gq to Gi switch in 5-HT_2A_R. We favor the hypothesis that dissociation of the CB_1_R-5HT_2A_R heteromer depends, in addition to the heteromeric interface, on the existence of additional direct interactions between intracellular receptor domains, as previously described for CB_1_R with adenosine and dopamine receptors.^38,39^ Those interactions might still remain after alteration of the TM heteromeric interface, and Gi coupling to 5-HT_2A_R remained unaltered.

It is important to note that there are similarities and differences between the predicted binding of the 3-point pharmacophore model, derived from the ^5.55^VYAYMYILW^5.63^ peptide, to the previously reported 5HT_2B_R-based homology model of 5HT_2A_R^6,16^ and the predicted binding mode of compounds **4**, **12**, and **31** to the recently released structure of 5HT_2A_R obtained by MD simulations. In both cases, the aromatic Ar2 element and the terminal phenyl ring of the ligands are placed in similar positions to interact with the Tyr254^5.58^, Phe329^6.41^, and Phe330^6.42^ cluster of aromatic rings. What remains different is the branched hydrophobic group of the ligands, which occupies the position of the aromatic Ar1 pharmacophoric element (W^5.63^ of the peptide), and the central aromatic heterocycle of **12** and **31**, or the phenyl ring of **4**, occupy the position of the hydrophobic HYD (L^5.62^ of the peptide). Moreover, the proposed hydrogen bond interaction of these ligands with Tyr254^5.58^ was not included in the pharmacophore model.

Tyr^5.58^ and the NPxxY and DRY motifs of class A GPCRs are responsible for opening the intracellular cavity, through the outward movement of TMs 5 and 6, for G protein binding.^40,41^ The binding mode of compounds **4**, **12,** and **31** was studied in the inactive conformations of TMs 5 and 6 of 5HT_2A_R, so the effect of the active conformations of these helices on their binding mode has not been evaluated. However, measurements of cAMP and pERK indicate that the binding of compounds **4**, **12,** and **31** to 5HT_2A_R does not affect receptor activation.

In conclusion, we believe compounds **4**, **12,** and **31** provide proof-of-principle for the design of optimized ligand-based disruptors of the CB_1_R-5HT_2A_R heteromer, opening new perspectives for *in vivo* studies.

## Experimental Section

### Peptide Synthesis, Analysis, and Purification

Peptides were assembled in a Prelude instrument (Protein Technologies, Tucson, AZ) running optimized Fmoc synthesis protocols as described before.^42^ Final deprotection and cleavage were performed with a CF_3_COOH/H_2_O/3,6-dioxa-1,8-octanedithiol/triisopropylsilane (94:2.5:2.5:1 v/v) cocktail for 90 min. Peptide analysis and purification were performed as previously detailed.^42^ Fractions of satisfactory purity (>90%) by analytical high-performance liquid chromatography (HPLC) were pooled, lyophilized, and analyzed for identity by HPLC–MS.

### Expression Vectors, HEK-293T Cell Culture, and Transient Transfection

Sequences encoding YFP Venus protein amino acid residues 1–155 and 156–238 were subcloned into the pcDNA3.1 vector to obtain the YFP Venus hemitruncated proteins. The human cDNAs for 5HT_2A_R and CB_1_R, cloned into the pcDNA3.1 were amplified and subcloned as described^6^ to give 5HT_2A_R-cYFP and CB_1_R-nYFP. Human embryonic kidney (HEK-293T) cells obtained from ATCC were grown in Dulbecco’s modified Eagle’s medium (DMEM) (Gibco) supplemented with 2 mM l-glutamine, 100 μg/mL sodium pyruvate, 100 U/mL penicillin/streptomycin, MEM nonessential amino acid solution (1/100), and 5% (v/v) heat-inactivated fetal bovine serum (FBS) (all supplements from Invitrogen, Paisley, Scotland, UK). Cells were maintained at 37 °C under an atmosphere of 5% CO_2_. HEK-293T cells were transfected with the corresponding fusion protein cDNA by the polyethylenimine (Sigma) method, as described before.^6,16^

### BiFC Assay

HEK-293T cells, after 48 h transient cotransfection with the cDNA encoding for 5HT_2A_R-cYFP and CB_1_R-nYFP (4 μg of cDNA for each construct), were treated with vehicle or 4 μM peptide or 4 μM ligand for 4 h at 37 °C. To quantify protein-reconstituted YFP Venus expression, cells (20 μg protein) were distributed in 96-well microplates (black plates with a transparent bottom, Porvair, King’s Lynn, UK), and emission fluorescence at 530 nm was read in a Fluo Star Optima fluorimeter (BMG Labtechnologies, Offenburg, Germany) equipped with a high-energy Xe flash lamp, using a 10 nm bandwidth excitation filter at 400 nm reading. Protein fluorescence expression was determined as the fluorescence of the sample minus the fluorescence of cells not expressing the fusion proteins (basal). Cells expressing 5HT_2A_R-cVenus and nVenus or CB_1_R-nVenus and cVenus showed similar fluorescence levels to nontransfected cells.

### cAMP Production and ERK-1/2 Phosphorylation Assays

For cAMP production, homogeneous time-resolved fluorescence energy transfer (HTRF) assays were performed as previously described.^6,16^ Cells (1000 cells/well) growing in medium containing 50 μM zardeverine were pretreated with the CB_1_R antagonist rimonabant (1 μM, RIM), and the 5-HT_2A_R antagonist MDL 100,907 (300 nM, MDL), or the corresponding vehicle in white ProxiPlate 384-well microplates (PerkinElmer, Waltham, MA, US) at 25 °C for 20 min and stimulated with the CB_1_R agonist WIN 55,212-2 (100 nM, WIN) and the 5HT_2A_R agonist DOI (100 nM, DOI) for 15 min before adding 0.5 μM FK or vehicle and incubated for an additional 15 min period. Fluorescence at 665 nm was analyzed on a PHERAstar Flagship microplate reader equipped with an HTRF optical module (BMG Lab Technologies, Offenburg, Germany). For ERK-1/2 phosphorylation assays, HEK-293 cells (30,000 cells/well) seeded in 96-well poly d-lysine-coated plates (Sigma-Aldrich, Madrid, Spain) were pretreated at 25 °C for 15 min with RIM (1 μM) and MDL (300 nM) or the corresponding vehicle and stimulated for an additional 7 min with WIN (100 nM) and DOI (100 nM). Phosphorylation was determined in white ProxiPlate 384-well microplates (PerkinElmer Life Sciences) by the α-screen bead-based technology using the amplified luminescent proximity homogeneous assay kit (PerkinElmer Life Sciences) and by using the Enspire multimode plate reader (PerkinElmer Life Sciences). Phosphorylation is expressed in arbitrary units, ALPHAcounts, as measured by light emission at 520–620 nm of the acceptor beads.

### Virtual Screening

A three-point feature pharmacophore was built using Discovery Studio 3.5 (Accelrys Software Inc., Discovery Studio Environment, Release 3.5, San Diego), using the reported experimental results obtained with different peptides and the previously reported computational model between TM5-Tat and a 5HT_2B_R-based homology model of 5HT_2A_R.^6,16^ Excluded space features were added by using the 5HT_2A_R homology model. Diverse conformations (250 per compound) of a subset of ∼4 M compounds, split according to heavy atom count in bins of 20–23, 24–26, 27–29, and 30–36, with molecular weight in the 250–500 Da range, *A* log *P* in the 2–7 range, and with <8 hydrogen bond acceptors, <4 hydrogen bond donors, and ≥2 aromatic groups, were rigidly fitted to the pharmacophore model. These solutions were scored from best to worst fitting (total score 0–3, with 3 being optimal) with the fit_value scoring function of Discovery Studio, which measures the spatial complementarity of the center of each group to the center of the feature for aliphatic (score 0–1) and combined spatial complementarity and directional for aromatic ring placement (score 0–1) in the two aromatic features. The top 300,000 compounds were further rescored using a flexible fit, in which slight flexibility is allowed for each conformation. Top-scoring 1000 compounds from each molecular size bin were clustered by similarity using DataWarrior.^43^ The process involves the calculation of a similarity matrix for each bin using fragment descriptors. The most similar compounds form the first cluster, and their similarity values are replaced by the mean similarity between the cluster center and the other compounds. This process of merging the most similar compounds or clusters, updating similarity values as weighted means, continues until the user-defined similarity threshold, which was set at 0.7, is reached. The highest scored compounds of each cluster were visually inspected when fitted in the pharmacophore model, while located in the defined pocket of the 5-HT_2A_R model, to finally select 41 diverse compounds. All compounds were purchased from different vendors with >95% purity.

### MD Simulations of Selected Compounds in Complex with 5HT_2A_R

The GPCRdb refined structure^44^ of inactive 5HT_2A_R (PDB id 7WC4)^27^ was used. The fusion protein was removed. Compounds **4**, **12**, and **31** were docked in a membrane-facing binding cavity between TMs 5 and 6 of 5HT_2A_R using rDock.^45^ Docking poses were ranked according to Stotal, and the poses in which the terminal phenyl ring of the ligands was placed between the group of aromatic rings of 5HT_2A_R formed by Tyr254^5.58^, Phe329^6.41^, and Phe330^6.42^ were initially selected (Figure S1). MD simulations of these complexes were performed using GROMACS,^46^ embedded in a rectangular lipid bilayer box, constructed using PACKMOL-memgen,^47^ containing 1-palmitoyl-2-oleoyl-*sn*-glycero-3-phosphocholine (POPC) and cholesterol (ratio 10:6), water molecules, and a 0.15 M concentration of monatomic Na+ and Cl– ions, using a previously described protocol.^48^ In particular, these initial systems were energy-minimized and subsequently subjected to various steps of MD equilibration (25 ns in total), where positional restraints on protein coordinates were progressively unreleased to accommodate the lipids and solvent around the protein. MD trajectories were produced at a constant temperature of 300 K by using separate v-rescale thermostats for the protein, ligand, lipids, and solvent molecules. In these simulations, a time step of 2 fs was used for the integration of the equations of motion. All bonds and angles were kept frozen by using the LINCS algorithm. Lennard-Jones interactions were computed by using a cutoff of 10 Å, and the electrostatic interactions were treated by using the particle mesh Ewald method. The amber14sb-ildn force field was used for the protein, solvent, and ions,^49^ lipid14 for lipids,^50^ and the general Amber force field (GAFF2) with HF/6-31G*-derived RESP atomic charges for the ligands.^51^ The analysis of the trajectories was performed using the Python package MDAnalysis,^52^ and solvent (SASA) and membrane-exposed (MESA) surface areas were calculated using FreeSASA.^53^

### Statistical Analysis

Experimental data were managed and analyzed with GraphPad Prism software, version 10 (San Diego, CA, USA) or IBM SPSS Statistics version 27.0 (IBM Corp., NY, USA). *P*-values lower than 0.05 were considered statistically significant.

## Supporting Information Available

ZINC id, log *P*, weight (kDa), and number of rotatable bonds of compounds **1**-**41** identified in the virtual screening exercise; statistical analyses used in signaling experiments; docking of compounds of compounds **4**, **12**, and **31** to 5HT_2A_R; computational model between compounds **40** and 5HT_2A_R; and NMR of compounds **4**, **12**, and **31** (PDF)
Molecular complexes of compounds **4**, **12**, and **31** with 5HT_2A_R (PDB, PDB, PDB)
SMILES of compounds **1**–**41** (CSV)

## Supplementary Material

jm4c01796_si_001.pdf

jm4c01796_si_002.pdb

jm4c01796_si_003.pdb

jm4c01796_si_004.pdb

## Abbreviations

5HT_2A_R: serotonin 5HT_2A_ receptor
BiFC: bimolecular fluorescence complementation
CB_1_R: cannabinoid CB_1_ receptor
CB_2_R: cannabinoid CB_2_ receptor
COM: center of mass
DOI: 2,5-dimethoxy-4-iodoamphetamine
MD: molecular dynamics
MDL: MDL100907
RIM: rimonabant
RMSD: root-mean-square deviation
THC: delta-9-tetrahydrocannabinol
TM: transmembrane
WIN: WIN 55,212-2
