# Supplementary material for: Design of Small Non-Peptidic Ligands That Alter Heteromerization between Cannabinoid CB1 and Serotonin 5HT2A Receptors
Source: J Med Chem. 2024 Dec 27;68(1):261–9. doi: 10.1021/acs.jmedchem.4c01796 (PMC11726681; doi:10.1021/acs.jmedchem.4c01796)

## Supporting information

# Design of Small non-Peptidic Ligands that Alter Heteromerization between Cannabinoid $CB_1$ and Serotonin $5HT_{2A}$ Receptors

Minos-Timotheos Matsoukas[1,#], Marc Ciruela-Jardí[2,3,#], Maria Gallo[4], Sergi Ferre[5], David Andreu[4], Vicent Casadó[3], Leonardo Pardo[2,*], and Estefanía Moreno[3,*]

[1]Department of Biomedical Engineering, University of West Attica, Ag. Spyridonos, Egaleo, 12243, Greece

[2]Laboratori de Medicina Computacional, Unitat de Bioestadística, Facultat de Medicina, Universitat Autònoma de Barcelona, 08193 Bellaterra, Spain

[3]Department of Biochemistry and Molecular Biomedicine, Faculty of Biology, Institute of Biomedicine of the University of Barcelona (IBUB), University of Barcelona, 08028 Barcelona, Spain

[4]Department of Medicine and Life Sciences (MELIS-UPF), Universitat Pompeu Fabra, 08003 Barcelona, Spain

[5]Integrative Neurobiology Section, National Institute on Drug Abuse, Intramural Research Program, National Institutes of Health, Baltimore, Maryland 21224, United States

[*]To whom correspondence should be addressed
Email: leonardo.pardo@uab.es, estefaniamoreno@ub.edu

**Table of Contents**

Table S1 .................................................... S3
Table S2 .................................................... S5
Figure S1 .................................................... S6
Figure S2 .................................................... S7
NMR spectra .................................................... S8

**Table S1.** Compounds **1**-**41** obtained in the Virtual Screening exercise of the ZINC20 database that were experimentally tested in BiFC assays.

| Number | ZINC ID | logP | Weight (kDa) | # rotatable bonds |
|---|---|---|---|---|
| 1 | ZINC000008074648 | 3.9075 | 309 | 6 |
| 2 | ZINC000012324308 | 4.2478 | 313 | 11 |
| 3 | ZINC000058184768 | 4.5072 | 335 | 9 |
| 4 | ZINC000004902363 | 4.6516 | 295 | 10 |
| 5 | ZINC000019793093 | 3.8287 | 314 | 7 |
| 6 | ZINC000004906251 | 3.8782 | 332 | 9 |
| 7 | ZINC000065605933 | 3.2092 | 288 | 10 |
| 8 | ZINC000012404787 | 5.5438 | 340 | 6 |
| 9 | ZINC000012382832 | 3.8889 | 310 | 7 |
| 10 | ZINC000008598990 | 5.1261 | 351 | 9 |
| 11 | ZINC000003252442 | 4.7422 | 334 | 9 |
| 12 | ZINC000002645797 | 4.7885 | 323 | 9 |
| 13 | ZINC000036349658 | 6.4771 | 389 | 8 |
| 14 | ZINC000067713635 | 3.3279 | 332 | 7 |
| 15 | ZINC000000092389 | 4.583 | 334 | 9 |
| 16 | ZINC000002195478 | 6.42548 | 359 | 8 |
| 17 | ZINC000005505999 | 4.6738 | 347 | 9 |
| 18 | ZINC000004789866 | 3.2698 | 337 | 9 |
| 19 | ZINC000032753650 | 4.6933 | 351 | 9 |
| 20 | ZINC000096343799 | 3.0973 | 337 | 9 |
| 21 | ZINC000004844985 | 4.5269 | 340 | 11 |
| 22 | ZINC000044085459 | 6.4057 | 377 | 7 |
| 23 | ZINC000095429525 | 3.5823 | 324 | 6 |
| 24 | ZINC000065393706 | 3.9998 | 343 | 7 |
| 25 | ZINC000037398243 | 4.5495 | 349 | 11 |
| 26 | ZINC000009267181 | 4.1349 | 348 | 9 |
| 27 | ZINC000005727162 | 5.32208 | 328 | 7 |
| 28 | ZINC000035562334 | 4.5783 | 349 | 10 |
| 29 | ZINC000037525736 | 5.5466 | 380 | 9 |
| 30 | ZINC000044895984 | 3.4823 | 335 | 9 |
| 31 | ZINC000036360428 | 4.1358 | 322 | 9 |
| 32 | ZINC000012785569 | 5.2243 | 375 | 9 |
| 33 | ZINC000097949683 | 6.0341 | 371 | 9 |
| 34 | ZINC000002708440 | 6.473 | 395 | 7 |

| | | | | |
|---|---|---|---|---|
| 35 | ZINC000100424414 | 5.3653 | 379 | 9 |
| 36 | ZINC000005545395 | 6.3784 | 352 | 7 |
| 37 | ZINC000000755201 | 5.2825 | 390 | 9 |
| 38 | ZINC000006736386 | 6.2603 | 402 | 11 |
| 39 | ZINC000036004541 | 5.0556 | 366 | 10 |
| 40 | ZINC000036041977 | 4.723 | 418 | 10 |
| 41 | ZINC000004783745 | 5.747 | 418 | 11 |

**Table S2.** Statistical analyses used in signaling experiments

| | Factor | p-value | Conclusions |
|---|---|---|---|
| 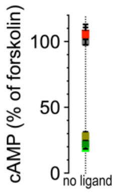  | treatment | <0.0001 | 5HT$_{2A}$Rago < FK (p<0.0001)<br>CB$_1$Rago < FK (p<0.0001)<br>5HT$_{2A}$Rago+CB$_1$Rago < 5HT$_{2A}$Rago (p=0.659)<br>5HT$_{2A}$Rago+CB$_1$Rago < CB$_1$Rago (p=0.930)<br>5HT$_{2A}$Rago+CB$_1$Ranta < FK (p=0.907)<br>CB$_1$Rago+5HT$_{2A}$Ranta < FK (p=0.953) |
| 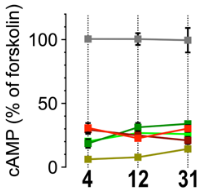  | treatment<br>ligand<br>interaction | <0.0001<br>0.239<br>0.075 | 5HT$_{2A}$Rago < FK (p<0.0001)<br>CB$_1$Rago < FK (p<0.0001)<br>5HT$_{2A}$Rago+CB$_1$Rago < 5HT$_{2A}$Rago (p<0.0001)<br>5HT$_{2A}$Rago+CB$_1$Rago < CB$_1$Rago (p<0.0001)<br>5HT$_{2A}$Rago+CB$_1$Ranta < FK (p<0.0001)<br>CB$_1$Rago+5HT$_{2A}$Ranta < FK (p<0.0001) |
| 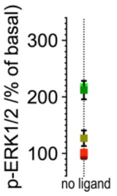  | treatment | <0.0001 | 5HT$_{2A}$Rago > basal (p<0.0001)<br>CB$_1$Rago > basal (p<0.0001)<br>5HT$_{2A}$Rago+CB$_1$Rago > 5HT$_{2A}$Rago (p>0.999)<br>5HT$_{2A}$Rago+CB$_1$Rago > CB$_1$Rago (p>0.999)<br>5HT$_{2A}$Rago+CB$_1$Ranta > basal (p=0.999)<br>CB$_1$Rago+5HT$_{2A}$Ranta > basal (p=0.999) |
| 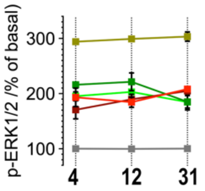  | treatment<br>ligand<br>interaction | <0.0001<br>0.677<br>0.06 | 5HT$_{2A}$Rago > basal (p<0.0001)<br>CB$_1$Rago > basal (p<0.0001)<br>5HT$_{2A}$Rago+CB$_1$Rago > 5HT$_{2A}$Rago (p<0.0001)<br>5HT$_{2A}$Rago+CB$_1$Rago > CB$_1$Rago (p<0.0001)<br>5HT$_{2A}$Rago+CB$_1$Ranta > basal (p<0.0001)<br>CB$_1$Rago+5HT$_{2A}$Ranta > basal (p<0.0001) |

One way or two-way ANOVA, followed by Tukey's multiple comparison tests, was used to analyse the data depicted in the left column

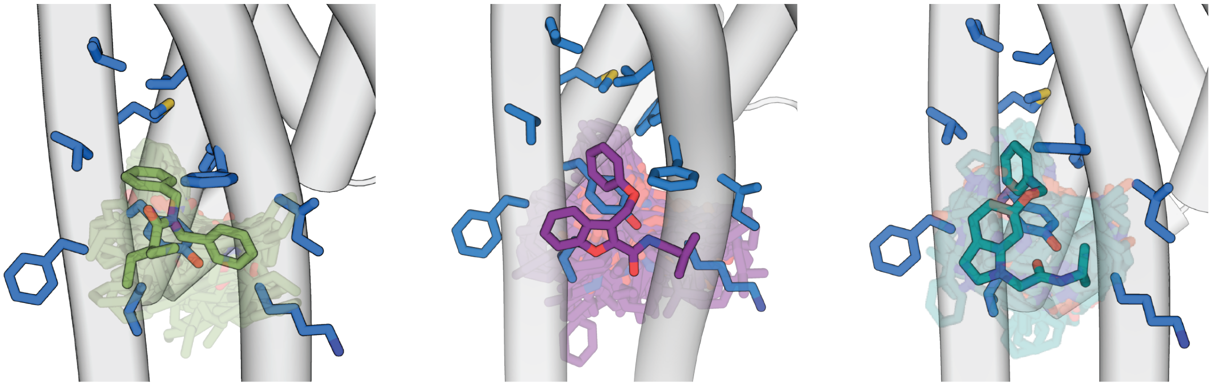

**4** **12** **31**

**Figure S1.** Superposition of the docking poses of compounds **4**, **12**, and **31** (light colored sticks) and the final predicted binding mode (dark colored sticks) in the membrane-facing cavity between TMs 5 and 6 (grey cylinders) of $5HT_{2A}R$. The amino acids predicted to contact the ligands are shown in blue sticks.

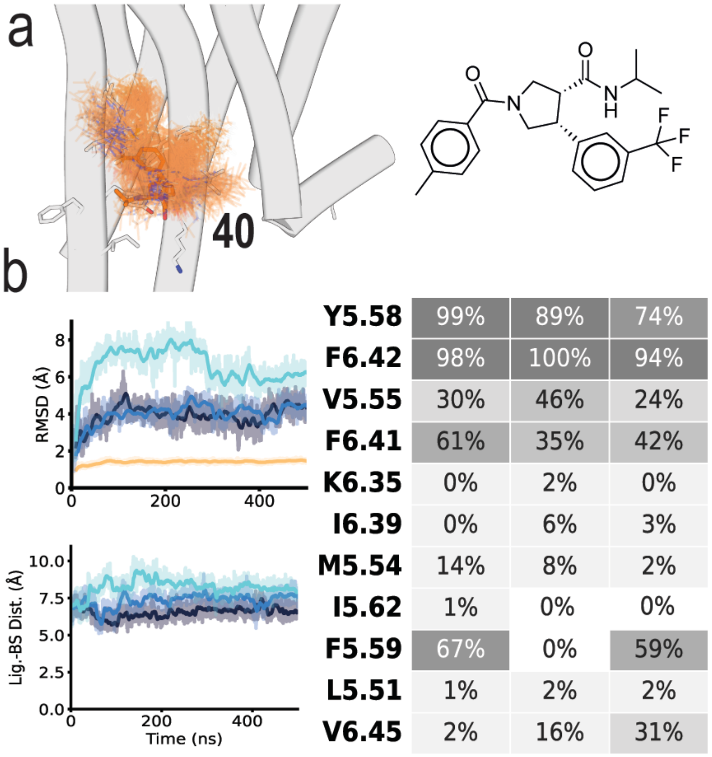


**Figure S2.** Computational model between inactive compound **40** (negative control) and $5HT_{2A}R$ (PDB id 7WC4). (a) Representative structure (solid sticks) and evolution (lines) of compound **40** (in orange) in complex with $5HT_{2A}R$ (gray cylinders, only the initial structure is shown) during MD simulations (one replica is displayed for better visualization although additional replicas showed consistent behavior). (b) The stability of the ligand-receptor complex was analyzed via root mean-square deviations (RMSD) of the ligand heavy atoms as devised from three replicas of unbiased 500 ns MD simulations (blue colours), and the stability of $5HT_{2A}R$ was analyzed via RMSD of the receptor Cα atoms (orange) (top panel). Evolution of the distance between the center of mass (COM) of the ligand and the residues in the binding site, along the trajectories (blue colours) (bottom panel). Detailed views and heatmaps (calculated with GetContacts, https://getcontacts.github.io/interactions.html) depicting the predicted interactions between compound **40** and $5HT_{2A}R$ during three replicas of unbiased 500 ns MD simulations.

NMR of compounds **4**, **12**, and **31** as provided by the vendors.

| | | | | | |
|---|---|---|---|---|---|
| 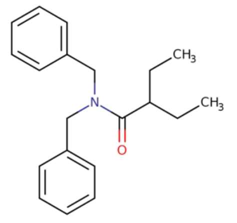 | **4** | ZINC000004902363 | Molport-001-500-854 | STK447807 | Vitas |

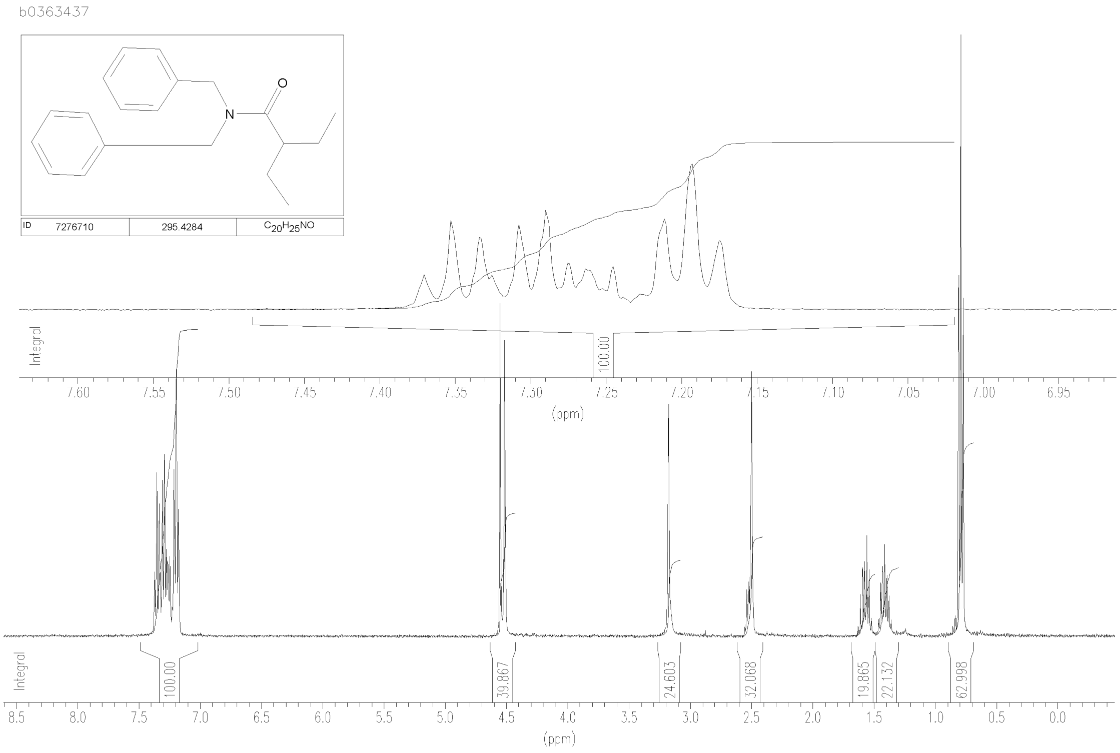

| | | | | | |
|---|---|---|---|---|---|
| 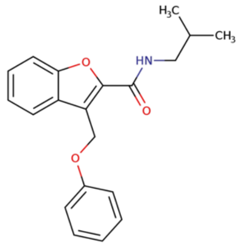 | **12** | ZINC000002645797 | Molport-004-061-257 | Z30868285 | Enamine |

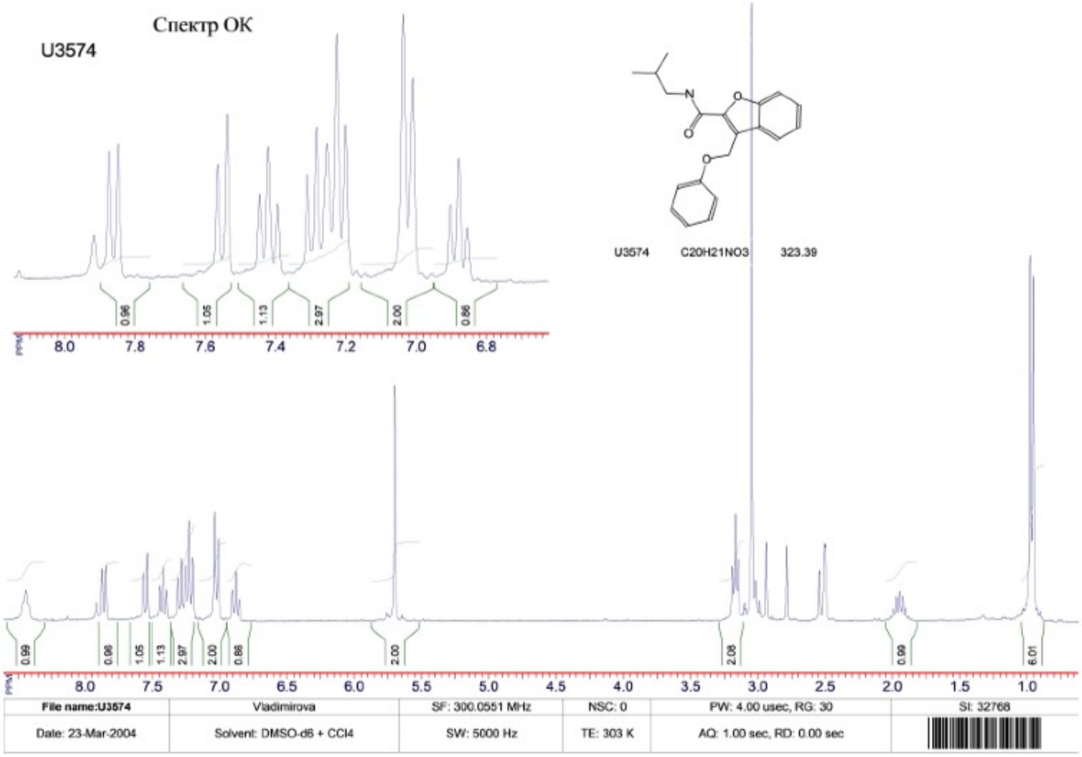

| | | | | | |
|---|---|---|---|---|---|
| 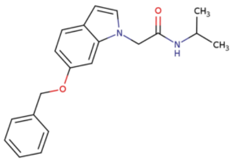 | **31** | ZINC000036360428 | Molport-008-320-659 | STK659895 | Vitas |

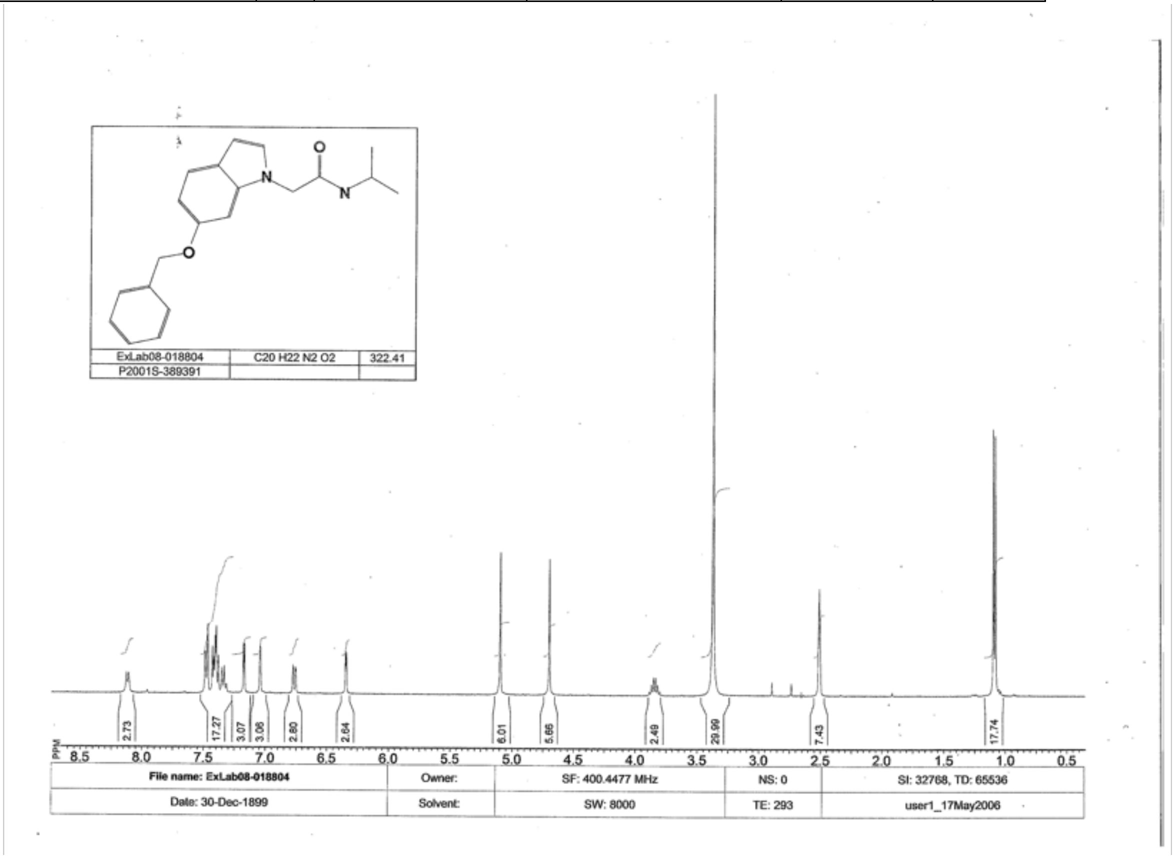

Supplement: Supplementary file 1 — jm4c01796_si_001.pdf [file jm4c01796_si_001.pdf]
